# Comparative Transcriptome Analysis Reveals Key Genes Related to Erythritol Production in *Yarrowia lipolytica* and the Optimization of Culture Conditions

**DOI:** 10.3390/ijms26094180

**Published:** 2025-04-28

**Authors:** Wei Fu, Ming Xu, Fan Yang, Xianzhen Li

**Affiliations:** School of Biological Engineering, Dalian Polytechnic University, Dalian 116034, China; fu18840808926@163.com (W.F.);

**Keywords:** erythritol production, *Yarrowia lipolytica*, mutagenesis, comparative transcriptome analysis, batch fermentation

## Abstract

Erythritol has been widely used in the food industry, which predominantly synthesizes it via microbial fermentation, in which *Yarrowia lipolytica* serves as the preferred candidate chassis strain. However, the wild-type strain of *Y. lipolytica* exhibits several limitations, including suboptimal industrial performance and elevated levels of by-products, which pose significant challenges in biomanufacturing processes. It is significant to understand the synthesis mechanism of erythritol for improving the capacity of erythritol production by *Y. lipolytica*. In this study, a mutant exhibiting high erythritol production and stable genetic performance was obtained via a combination of UV and atmospheric and room-temperature plasma mutagenesis. Some key genes related to erythritol production were identified through comparative transcriptome analysis of the mutant strain, revealing significant changes in their expression levels. Individual overexpression of the genes encoding ribose-5-phosphate isomerase, glucose-6-phosphate-1-epimerase, adenylate kinase, and alcohol dehydrogenase in *Y. lipolytica* Po1g enhanced erythritol production, demonstrating the critical role of each gene in erythritol production. This finding elucidates the molecular mechanism underlying the improved erythritol yield in the mutant strain. The *Y. lipolytica* mutant C1 produced 194.47 g/L erythritol in a 10 L fermenter with a productivity of 1.68 g/L/h during batch fermentation, surpassing the wild-type strain and reducing the cultivation time by 21 h. It is significant to understand the mechanism of erythritol synthesis for improving erythritol production and its application in industrial-scale production.

## 1. Introduction

Erythritol is a natural compound widely found in fruit with a high sugar content, seaweed, and mushrooms [1], whereas the principal method for erythritol production resides in microbial fermentation. Emerging studies on the physiological functions of erythritol highlight its benefits, stimulating growing interest in its development [2,3]. Erythritol is favored in the food industry, owing to its low-calorie property and non-participation in the Maillard reaction. And its high tolerance capacity and lack of toxic side effects render erythritol significant in the domains of medicine and healthcare [4,5,6,7]. At present, the rising prevalence of obesity-associated diseases has underscored the urgent need to reduce sugar intake. Consequently, the confectionery industry is increasingly developing low-sugar or sugar-free alternatives to meet the demands of health-conscious consumers. Erythritol, a virtually calorie-free polyol, serves as a functional sweetener for individuals afflicted with obesity and diabetes, as well as those seeking to modify their dietary habits [8]. Erythritol has become a widely recognized functional ingredient for human consumption, resulting in an application in multiple domains, such as food, pharmaceutical, cosmetic, and chemical, thereby highlighting its extensive potential utilization scenarios [7,8,9,10]. Previous explorations on erythritol production have predominantly focused on identifying high-productivity yeasts, including *Trigonopsis*, *Candida*, *Yarrowia*, *Pichia*, and *Pseudozyma* [11,12,13,14]. Notably, *Y. lipolytica* has become a preferred industrial species for erythritol production because of its ability to withstand high osmotic pressure, the availability of comprehensive gene editing tools, and its flexibility in substrate utilization [15]. In industrial production, the batch fermentation process of *Y. lipolytica* indicates remarkable bioprocess advantages, where the simplified design of single-batch feeding and the absence of nutrient supplementation significantly reduces operational complexity. The hyperosmotic medium system commonly employed in industrial practice utilizes glucose as the primary carbon source (200–250 g/L), supplemented with inorganic salts to maintain osmotic equilibrium. Mechanically agitated bioreactors are typically configured for erythritol production, with real-time monitoring of temperature, pH, and dissolved oxygen levels. Dissolved oxygen concentration is dynamically regulated by adjusting the agitation speed and aeration rates through variable-frequency agitation, ensuring the efficient and stable bioproduction of erythritol. Erythritol is predominantly generated in *Y. lipolytica* through the pentose phosphate pathway (PPP), in which erythrose-4-phosphate (E4P) is synthesized as a precursor of erythritol, followed by the conversion of E4P into erythrose catalyzed by erythrose-4-phosphate phosphatase; the resulting erythrose is reduced to erythritol via NADPH-dependent erythrose reductase [7,16]. Currently, *Y. lipolytica* has been established as a promising microbial platform for erythritol and other natural products via fermentation processes [17,18,19,20].

However, there are still some challenges in achieving high-yield erythritol production with wild-type strains, such as weak synthetic metabolism, excessive by-products, and suboptimal industrial performance [15]. As a solution, mutagenesis breeding provides a powerful approach to generate strains with favorable characteristics. For instance, *Streptomyces noursei* irradiated with UV showed significantly increased polyfungin B production along with enhanced chemical and biological activity compared to its wild-type counterpart [21]. Mironczuk et al. used UV mutagenesis to obtain a *Y. lipolytica* MK1 mutant with enhanced erythritol production efficiency, higher yield, and reduced by-product formation [22]. Compared with UV mutagenesis, atmospheric and room-temperature plasma (ARTP) mutagenesis exhibits higher efficacy in inducing DNA damage, activating cellular SOS repair systems, and generating stable mutants [23]. Consequently, ARTP mutagenesis provides a greater probability of obtaining positive mutants [24,25,26,27]. It has been reported that the *Brevivacillus* sp. mutant generated by ARTP exhibits enhanced antimicrobial activity against MRSA [28]. When *Saccharomyces boulardii* was subjected to ARTP mutagenesis, both selenium accumulation capacity and biomass yield were significantly increased, enhancing its applicability in the food and pharmaceutical industries [29]. Moreover, the combination of ARTP with UV mutagenesis demonstrates higher positive mutation rates and improved mutational efficiency compared to single-mutagenesis methods [30,31]. However, the stochastic nature of mutagenesis hinders the elucidation of the mechanism underlying enhanced erythritol production, resulting in insufficient theoretical support for data-driven microbial engineering. Recent studies indicate that multi-omics technologies provide a systematic framework to guide chassis cell optimization, thereby facilitating the development of high-yield industrial strains [32,33]. High-yield α-humulene production in *Y. lipolytica* was achieved through integrated transcriptome analysis and metabolic engineering [34]. Comparative transcriptome data helped to achieve an over-accumulation of menaquinone-7 by the *Bacillus subtilis* mutant [35]. Comparative transcriptome analysis was conducted to identify the crucial metabolic pathways and genes associated with tomato carotenoid biosynthesis for high lycopene production [36]. This integrated approach, combining random mutagenesis with multi-omics analysis, represents a promising strategy for developing high-performance microbial chassis, thereby advancing erythritol biosynthesis research. In addition to strain modification, the optimization of fermentation conditions serves as a critical step to enhance erythritol production in microbial strains. Precise regulation of medium components, such as carbon-to-nitrogen ratio adjustments and trace element supplementation, significantly affected the synthesis of the target product in the strain [18,19]. Moreover, integrating statistical experimental designs, such as response surface methodology (RSM), can elucidate nonlinear interactions among multiple variables [17], thereby establishing highly robust scale-up process models. The systematic optimization of cultivation parameters constitutes a core strategy for improving erythritol synthesis efficiency by dynamically balancing cellular metabolic activity with environmental stress, thereby enhancing industrial production efficiency.

In this study, *Y. lipolytica*, which has been widely adopted as the primary industrial strain for erythritol synthesis, owing to its well-characterized genetic background and advanced genetic engineering tools [37], was utilized as the parental strain to develop a high-yield mutant with stable genetic traits through ARTP and UV mutagenesis. The mutant was subsequently subjected to transcriptomic profiling to elucidate the molecular mechanisms underlying enhanced erythritol biosynthesis, thereby enabling the identification of effective metabolic engineering targets. Subsequent metabolic engineering in the model strain Po1g will involve the overexpression of these key genes to validate the effectiveness of transcriptomics-guided strain modification and confirm the critical role of each key gene in facilitating high erythritol production in mutant C1. Distinct from the single strategy of targeted genetic modifications to validate gene functions in traditional studies [38], this study will elucidate the molecular mechanisms underlying enhanced erythritol biosynthesis in mutant C1 through integrated transcriptomic analysis combined with systematic metabolic engineering in the model strain, highlighting the role of each key gene in the erythritol production pathway confirmed by transcriptomics, thereby establishing a closed-loop “composite mutagenesis–strain validation” workflow. Moreover, in contrast to prior studies concentrating on laboratory significance, our approach will prioritize industrial applicability. Batch fermentation in a fermenter with mutant C1 and the wild-type strain will validate the mutant-enhanced production stability and process compatibility. If the mutant exhibits reduced phenotypic variability, this will indicate its potential for robust scalability in industrial-scale production. This work provides a systematic understanding of erythritol biosynthesis through integrated transcriptomic analysis, systematic metabolic engineering in the model strain, and process parameter correlations. This systematic characterization establishes a foundation for the future metabolic engineering of high-yield erythritol-producing strains and provides a framework for constructing efficient microbial cell factories for analogous bioproduction applications. Moreover, the optimization of fermentation conditions establishes the foundation for efficient erythritol biosynthesis.

## 2. Results and Discussion

### 2.1. Mutation of Y. lipolytica for Improving Erythritol Production

**Figure 1:**
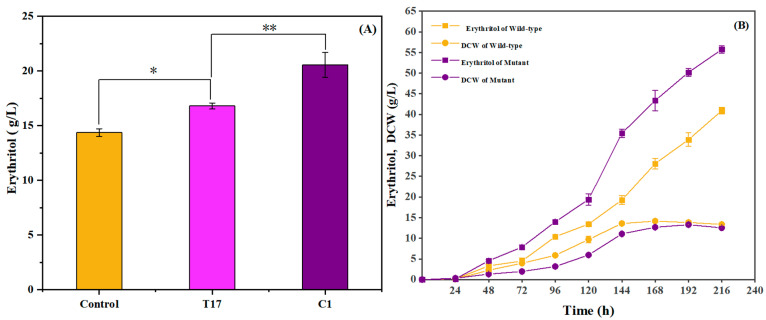
Erythritol production and dry cell weight (DCW) of the mutant strains compared to those of the wild-type. (**A**) Comparison of erythritol production capacity between mutant strains and the wild-type strain of *Y. lipolytica*. Note: data are presented as mean ± SD (n = 3). Statistical significance was determined by Student’s *t*-test (* *p* < 0.05; ** *p* < 0.01). (**B**) Time-course of erythritol production and DCW of both the wild-type strain and mutant C1. Yellow squares: Erythritol production (g/L) of the wild-type strain; Yellow circles: DCW (g/L) of the wild-type strain; purple squares: Erythritol production (g/L) of the mutant C1; purple circles: DCW (g/L) of the mutant C1.

To obtain a mutant producing a high yield of erythritol, both UV and ARTP mutagenesis were used for random mutation in the wild-type *Y. lipolytica* to improve erythritol production. A period of 90 s for UV mutagenesis and a period of 180 s for ARTP mutagenesis were selected as the ideal exposure durations because efficient mutagenesis was obtained when the mortal ratio was over 90% [39]. After mutation was induced by UV irradiation, a convenient high-throughput screening method based on red colony identification on TTC plates was applied to select erythritol-producing mutants. A total of 1200 mutants having stronger color intensity were recognized when compared with the colony of the wild-type strain, suggesting a potential increased erythritol production in those mutants. To further confirm the erythritol production by those mutants picked out from the TTC plate, the mutant with red color colonies was cultured in a 24-well plate, and the mutant producing erythritol was recognized by the TLC assay. A total of 160 mutants exhibiting enhanced erythritol production were identified through the TLC assay. Subsequently, the capacity of producing erythritol was quantitatively assessed with the HPLC assay. One UV-mutated *Y. lipolytica* strain, named T17, exhibited strikingly increased erythritol production compared to the wild-type strain. To further improve its capability of producing erythritol for scale-up to the industrial level, an enhanced ARTP mutagenesis was executed on mutant T17. One high-yield mutant was detected and named C1. To evaluate enhanced erythritol production in mutants, mutants T17 and C1 were cultured in 50 mL flasks containing 15 mL of high osmotic pressure screening medium under fermentation conditions (30 °C, 200 rpm) for 144 h. Erythritol production was subsequently quantified using HPLC. As shown in Figure 1A, the erythritol production of mutant C1 was increased by 22% when compared with UV-mutated mutant T17 and by 42% when compared with the wild-type *Y. lipolytica*. Subsequently, mutant C1 was subjected to serial subculturing, and erythritol production was quantified by HPLC at each generation to evaluate its genetic stability. Mutant C1 maintained a stable level of erythritol production over ten generations, showing no statistically significant production decline (*p* > 0.05), which suggested that this mutant not only manifested a considerable capacity to produce erythritol but also exhibited genetic stability.

The cultures were prepared in 250 mL flasks containing 30 mL of initial fermentation medium, and the dynamic profiles of biomass accumulation and erythritol biosynthesis were systematically tracked throughout the fermentation process, with temporal trends comprehensively illustrated in Figure 1B. When compared with the wild-type *Y. lipolytica*, mutant C1 could produce a higher yield of erythritol but gave a lower biomass and underwent retarded growth, especially in the early stage of fermentation. Such a phenomenon was probably ascribed to stronger proliferation and enhanced nutrient absorption by a certain strain, which presumably resulted in an increased availability of precursors essential for synthesizing other secondary metabolites [40,41,42,43]. Thus, the elevated biomass might not exert a beneficial influence on the accumulation of the target product. Recent studies have also illustrated that increased erythritol production is frequently associated with cell growth attenuation [44]. It was presumed that the mutation might transform the metabolic pathway of the mutated strain, which reallocated more energy and resources toward erythritol synthesis rather than facilitating cell growth, thereby leading to a relatively low biomass yield but a high erythritol yield. Moreover, foam formation by mutant C1 during fermentation was much less than that by wild-type *Y. lipolytica*, which confers a significant benefit to industrial applications. Thus, mutant C1 was selected for the following studies.

### 2.2. Comparative Transcriptome Analysis

**Figure 2:**
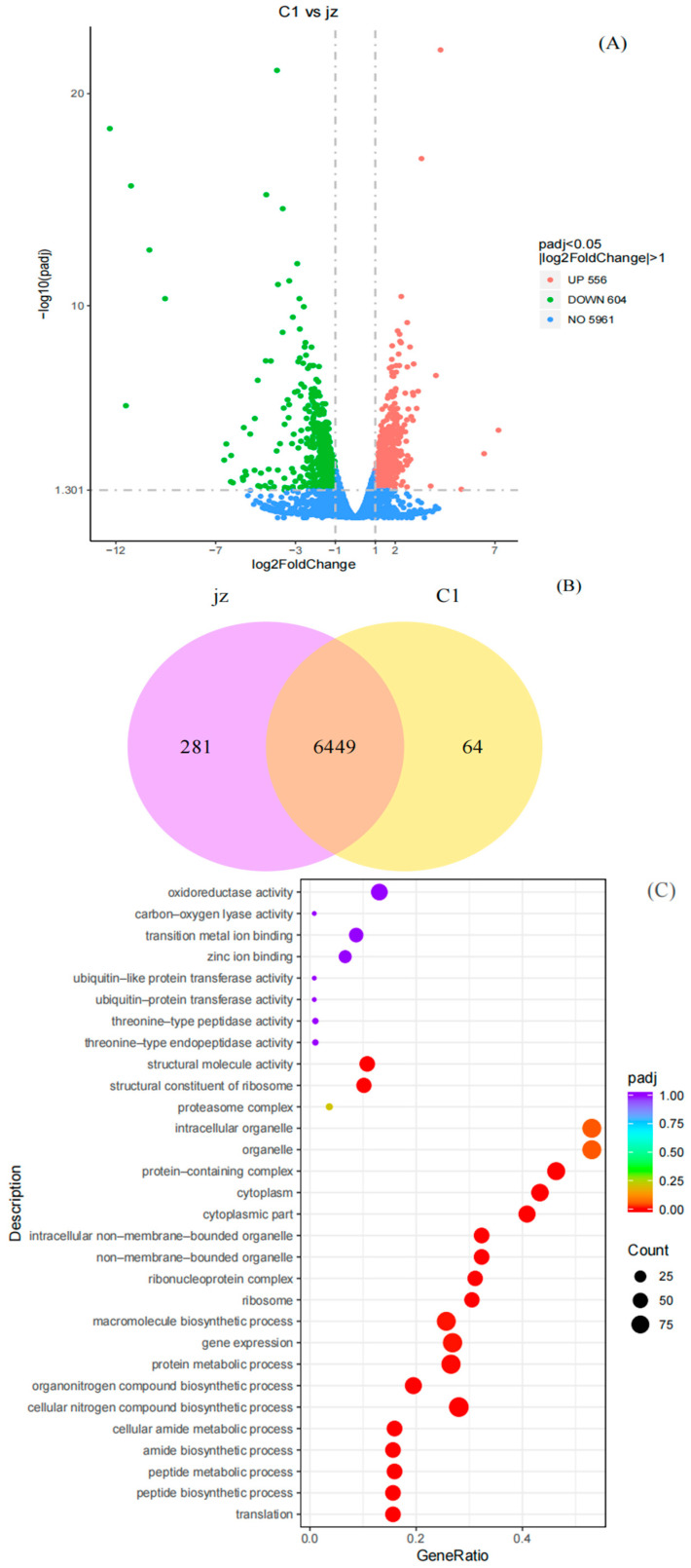

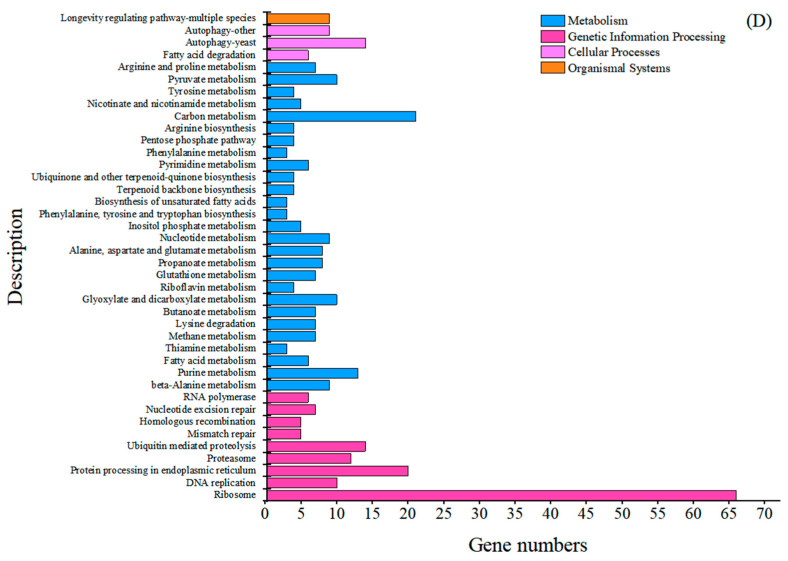
Comparative transcriptome analysis by RNA-seq reveals differential gene expression between mutant C1 and its wild-type strain in *Y. lipolytica*. (**A**) Volcano plot of differentially expressed genes (DEGs), in which red dots depict up-regulated DEGs, green dots depict down-regulated DEGs, and blue dots represent non-significant genes. The *X*-axis depicts the multiple of difference after log2 transformation, and the *Y*-axis depicts the significance value after −log10 transformation. (**B**) Venn diagram analysis identifying 6449 shared genes between the wild-type and mutant, with 64 genes unique to the mutant and 281 genes exclusive to the wild-type. (**C**) Enrichment map of the top 30 Gene Ontology (GO) terms ranked by the lowest padj, categorized into three domains: biological process (BP), molecular function (MF), and cellular component (CC). The bubble size corresponds to the number of DEGs per term, the *X*-axis is the enrichment ratio, and the *Y*-axis is the GO term, the significance of gene set enrichment is visualized through a color gradient (deeper red indicates lower adjusted *p*-values, padj) (**D**) KEGG pathway enrichment analysis of DEGs identifying the top 40 significantly enriched pathways, categorized into four functional classes according to KEGG Brite Hierarchy: metabolism, Genetic Information Processing, Cellular Processes, and Organismal Systems.

To understand why the mutated *Y. lipolytica* shows augmented productivity and to identify the factor accountable for this enhancement, comparative transcriptome sequencing of both wild-type strain CICC 33063 and mutant C1 was performed by the RNA-seq assay on the Illumina Nova Seq 6000 platform (Novogene Co., Ltd., Beijing, China) The raw data garnered from the RNA-seq assay were subsequently submitted to the NCBI SRA database (ID: PRJNA975267), and a comparison of the mRNA expression level between the two strains was implemented, grounded on the transcriptome analysis. As presented in Figure 2A, the volcano plot revealed that a total of 1160 genes were differentially expressed in the mutant, among which 604 genes were down-regulated and 556 genes were up-regulated. The Venn diagram in Figure 2B shows the differentially expressed genes (DEGs) between the two strains, in which there are 6449 shared genes along with 281 genes exclusive to the wild-type strain and 64 genes specific to the mutant. These data reveal significant differences in the gene expression levels of the mutant compared to its parent strain (*p* < 0.05). Subsequently, Gene Ontology (GO) and Kyoto Encyclopedia of Genes and Genomes (KEGG) analyses were executed for a comprehensive functional enrichment assessment to further illuminate those DEGs. In the GO analysis, pronounced discrepancies were detected in the DEGs related to cellular components (CCs), biological processes (BPs), and molecular functions (MFs). As shown in Figure 2C, specific modifications were observed in cell membrane structures and organelles, including both non-membrane-bound organelles and outer membranes. Prior studies have illustrated that mutagenic factors can induce mutations in genes encoding membrane-associated proteins, thereby altering the functionality of membrane structures and organelles, ultimately leading to adaptive responses to mutagenic stimuli [45]. The enriched differentially expressed genes (DEGs) were further analyzed through KEGG pathway annotation. Based on the KEGG classification system, the top 40 enriched DEGs were categorized into four functional modules: metabolism, Genetic Information Processing, Cellular Processes, and Organismal Systems. The KEGG pathway enrichment analysis demonstrated that the highest number of DEGs were enriched in the ribosome, which is closely associated with protein translation.

### 2.3. Key Genes Affecting Erythritol Production in Oxidative Synthesis

**Figure 3:**
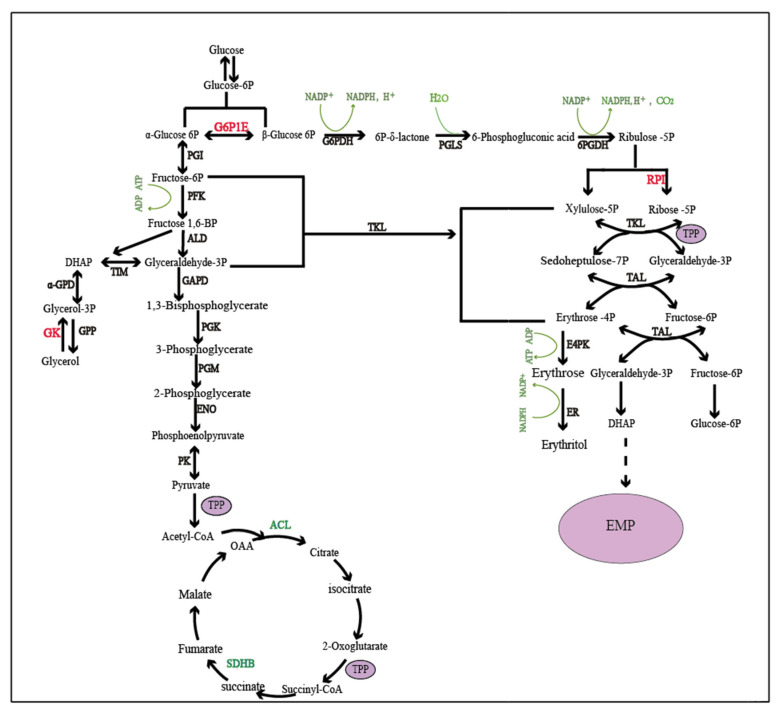
Schematic overview of the genes involved in the relevant pathways of erythritol production. HK, hexokinase; G6P, glucose-6-phosphatase; G6PDH, glucose-6-phosphate dehydrogenase; PGLS, 6-phosphogluconolactonase; 6PGDH, 6-phosphogluconate dehydrogenase; PGI, phosphoglucose isomerase; GK, glycerol kinase; PFK, Phosphofructokinase; TKL, transketolase; TAL, transaldolase; TIM, Triose phosphate isomerase; GAPD, glyceraldehyde-3-phosphate dehydrogenase; E4PK, erythrose-4-phosphate kinase; PGK, phosphoglycerate kinase; PGM, phosphoglycerate mutase; ENO, neuron-specific enolase; ER, erythritol reductase; GPP, 3-phosphoglycerine esterase; α-GPD, α-glycerophosphate dehydrogenase.

Central carbon metabolism comprises the Embden–Meyerhof–Parnas (EMP) pathway, the tricarboxylic acid (TCA) cycle, and the pentose phosphate pathway (PPP), which provide biochemical precursors and the energy supply for cells [46]. As shown in Figure 3, erythritol was synthesized in the PPP, including both oxidative and non-oxidative synthesis [7]. Comparative transcriptome analysis also suggested that the significant difference at the gene expression level occurred in those metabolic pathways. As shown in the TCA cycle (Figure 3), the succinate dehydrogenase (ubiquinone) iron-sulfur subunit (SDHB) is responsible for catalyzing the conversion of succinic acid to fumaric acid [47], and ATP-citrate lyase (ACLY) is responsible for catalyzing the conversion of citric acid to acetyl-CoA [48]. The expression level of those two genes in mutant C1 was significantly down-regulated to 0.28-fold and 0.4-fold of the control, respectively, which resulted in an inhibition of the oxidation of pyruvate entering the TCA cycle and suppressing the normal working of the EMP-TCA metabolic pathway. Considering the competitive carbon flux of glucose in the central metabolic pathway [49], such down-regulation of these genes likely shifted carbon flux away from the TCA cycle, redirecting it to alternative glucose metabolism pathways. This led to sufficient carbon flux availability, which supported erythritol production. Thus, these two down-regulated genes in the TCA cycle were selected as the key genes affecting erythritol production.

As shown in Figure 3, Glucose-6-phosphate-1-epimerase (G6P1E) catalyzes the interconversion between the α- and β-anomeric configurations of glucose-6-phosphate (G6P) [50,51]. Although such isomerization is a spontaneously reversible reaction, the conversion rate from α-G6P to β-G6P is higher than that from the reverse reaction [52], and β-G6P can be transformed into 6-phosphogluconate-δ-lactone (6-PGL), catalyzed by glucose-6-phosphate dehydrogenase (G6PDH), which serves as the initial rate-limiting step in the PPP [52,53]. The expression of G6P1E in mutant C1 was up-regulated 2.35-fold, which increased G6P isomerization and alleviated the rate-limiting step. Thus, the up-regulation of G6P1E potentially enhances erythritol production due to the added carbon flux being channeled into the PPP. G6P1E is regarded as a crucial gene influencing erythritol production.

The expression level of ribose-5-phosphate isomerase (RPI) was also detected to be up-regulated by 2.87-fold, which catalyzes the conversion of ribulose-5-phosphate into ribose-5-phosphate (R5P) in the PPP, and was assumed to play a crucial role in erythritol synthesis. It was reported that the overexpression of subsequent transketolase and transaldolase in the PPP has almost no impact on erythritol synthesis because of an imbalanced supply of R5P [54]. So, the up-regulated RPI expression in the mutant could expedite the conversion of ribulose-5-phosphate and R5P, providing abundant and balanced substrate for the subsequent transketolase and transaldolase reactions, thereby increasing erythritol production [54]. Sua’stegui et al. also concluded that the overexpression of RPI was a feasible approach for redirecting carbon flux from EMP to E4P biosynthesis in *Saccharomyces cerevisiae*, thus boosting the production of drug precursors [55]. Hence, RPI was also regarded as a key gene affecting the erythritol yield.

Carbon flux toward TCA was attenuated due to the down-regulation of both SDHB and ACLY. In contrast, the up-regulation of G6P1E expression could increase G6P isomerization, which relieved the initial rate-limiting step in the PPP. Thereby, the carbon flux could be channeled into the PPP from TCA because of the changes in the expression of genes encoding SDHB, ACLY, and G6P1E. Moreover, the up-regulation of RPI not only plays a significant role in the augmentation of erythritol production but also is a feasible approach to shift carbon flux toward the PPP. As shown in Figure 3, redirecting carbon flux to the PPP is beneficial for erythritol production because the PPP is the principal pathway for synthesizing erythritol. Gene expression associated with both EMP and TCA constitutes a vital indicator of capacity for microbial viability in aerobic microbes because the substantial generation of free energy in TCA is necessary for cell growth [32]. The difference in the gene expression described above suggested that a larger proportion of carbon flux was channeled into the principal synthetic route for erythritol production but not into the route for cell growth in the mutant, which resulted in an increasing erythritol yield accompanied by reduced biomass. This is consistent with an efficient strategy for enhancing erythritol production by restraining cell growth and channeling additional carbon sources into the PPP [54].

### 2.4. Key Genes Affecting Erythritol Production in Non-Oxidative Synthesis

As shown in Figure 3, glycerol was phosphorylated into glycerol-3-phosphate (G3P) by glycerol kinase, followed by a dehydrogenation catalyzed by 3-phosphoglycerol dehydrogenase to form dihydroxyacetone phosphate (DHAP). Finally, DHAP was isomerized to 3-phosphoglyceraldehyde (GA3P) by phosphoglycerate isomerase (TPI) and into EMP [56,57]. E4P was also produced by the transketol reaction between GA3P and F6P, catalyzed by transketolase [58]. In this study, the expression level of glycerol kinase (GK) was detected to be up-regulated by 2.75-fold in the mutant, which is more favorable for glycerol consumption and ultimately E4P generation through a non-oxidative pathway. The overexpression of GK was found to stimulate erythritol synthesis in the model strain Po1f [54]. Carly et al. also reported that the coexpression of GK and TKL in *Y. lipolytica* gave rise to an augmented capacity for glycerol uptake and a notable escalation in the erythritol conversion rate [58]. Thereby, GK was regarded as an important gene affecting erythritol production.

Thiamine pyrophosphate (TPP), as an active form of thiamine, is a cofactor for many key enzymes involved in metabolic pathways that are crucial for cell growth [59]. Thiamine deficiency exerts a considerable influence on central carbon metabolism and the level of ATP synthase in the respiratory chain [60]. The thiamine metabolic pathway could be inferred to play an important role in cell growth and metabolism. It was observed that the expression level of the gene encoding adenylate kinase (AK) was up-regulated by 2.49-fold in mutant C1. AK, with increased expression in the thiamine metabolism pathway, potentially stimulates erythritol production. Thus, AK could be considered as a key gene affecting erythritol production. *Y. lipolytica* produces citric acid as a by-product during erythritol biosynthesis via glucose fermentation [61]; the accumulation of citric acid and other organic acids can lead to medium acidification, which may inhibit erythritol biosynthesis. Notably, alcohol dehydrogenase 2 (ADH2) regulates respiratory and metabolic functions by modulating aerobic respiration and mitochondrial activity, making it a promising target for enhancing organic acid tolerance [62]. Thus, it is hypothesized that overexpression of ADH2 effectively mitigates acidic stress by stabilizing intracellular pH, thereby improving cell growth and metabolic processes. Previous studies illustrated that the overexpression of ADH1 (arabinitol dehydrogenase) in *Y. lipolytica* reduces arabinitol formation and redirects carbon flux toward erythritol synthesis, achieving higher carbon conversion efficiency [63]. Intriguingly, ADH2 exhibits broader substrate specificity, including arabinitol, xylitol, sorbitol, and mannitol. This versatility suggests that ADH2 overexpression could similarly minimize metabolic competition and enhance erythritol productivity by alleviating arabinitol formation. Thus, the ADH2 gene, exhibiting a 4.92-fold up-regulation in mutant C1, has been identified as an indispensable gene affecting erythritol production. In the fatty acid metabolic pathway, the gene expression levels of both acyl-CoA oxidase (ACOX) and enoyl-CoA hydratase (ECOH) were up-regulated 3.07-fold and 2.18-fold, respectively. It is known that energy is of paramount importance for cell growth and development, which might be supplied by fatty acid catabolism [64]. It was presumed that those two up-regulating genes impacted the energy requirement for microbial growth and exerted an influence on both cell growth and metabolic processes. The identification of those key genes might offer target genes for genetic modification with metabolic engineering methodology, which potentially directs the establishment of efficient cell factories.

### 2.5. Validation of Key Genes Affecting Erythritol Production by RT-qPCR Expression Analysis

**Figure 4:**
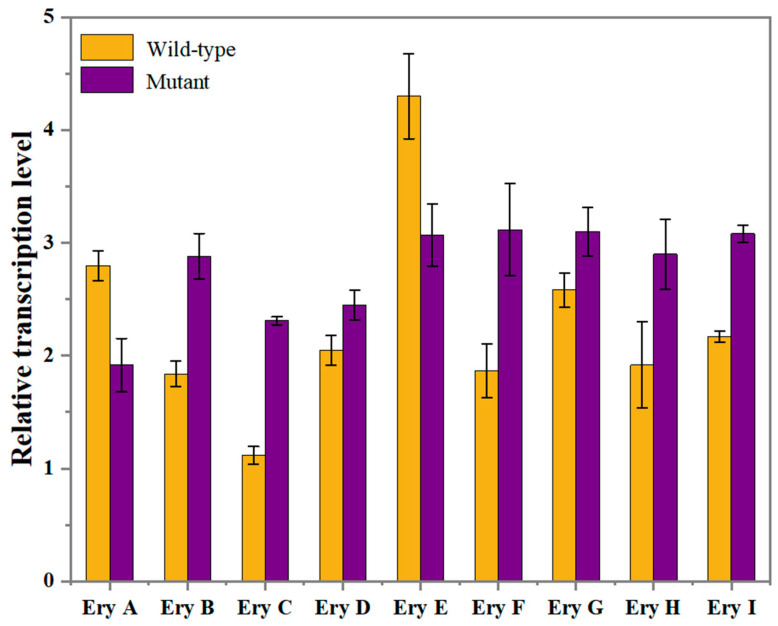
Validation for the expression level of key genes affecting erythritol production in *Y. lipolytica* by RT-qPCR. The genes encode succinate dehydrogenase (ubiquinone) iron-sulfur subunit (EryA), glucose-6-phosphate-1-epimerase (EryB), ribose-5-phosphate isomerase (EryC), glycerol kinase (EryD), ATP citrate-lyase (EryE), adenylate kinase (EryF), acyl-CoA oxidase (EryG), alcohol dehydrogenase (EryH), and enoyl-CoA hydratase (EryI).

Some key genes were identified to be variable at the gene expression level relevant to erythritol synthesis based on the comparative transcriptome analysis, as described above. To verify the accuracy of the transcriptome sequencing results, RT-qPCR validation was carried out on those genes with modulated expression levels. As shown in Figure 4, the overall trend of expression alteration complies with the transcriptomic data, although the expression levels of those genes might fluctuate, which strongly highlights the significance of changes in the expression level of those genes to promote erythritol production. Moreover, considering that the PPP is a pathway that produces erythritol, an increase in the expression level of genes affiliated with the PPP could potentiate metabolic flux into this pathway [65,66]. And it was also reported that the intensified expression of a key enzyme in the PPP with glucose as substrate was an efficacious tactic for yeast to yield a high added-value product [67]. In accordance with the RT-qPCR analysis (Figure 4), a significant up-regulation of both RPI and G6P1E in PPP was detected at the gene transcription level, which presumably facilitated carbon flux toward the PPP and thereby potentially enhanced the synthesis of substances related to the PPP. Thus, to enhance erythritol production, it is important to aim at fortifying the PPP by incrementing gene expression, thus guaranteeing an efficient carbon flux supply to the PPP. Thus, the first step for the following metabolic engineering improvement is to overexpress those key genes directly associated with the PPP. Simultaneously, the AK-encoding gene was observed to be up-regulated at the transcription level. Given the significance of the thiamine metabolic pathway for erythritol production, AK functions as a vital target gene in metabolic engineering as well. The ADH2-encoding gene was also observed to be up-regulated at the transcription level. Given the importance of ADH2 in the above analysis, we also consider ADH2 as an important target gene in metabolic engineering.

### 2.6. Rational Metabolic Engineering for Improving Erythritol Production

**Figure 5:**
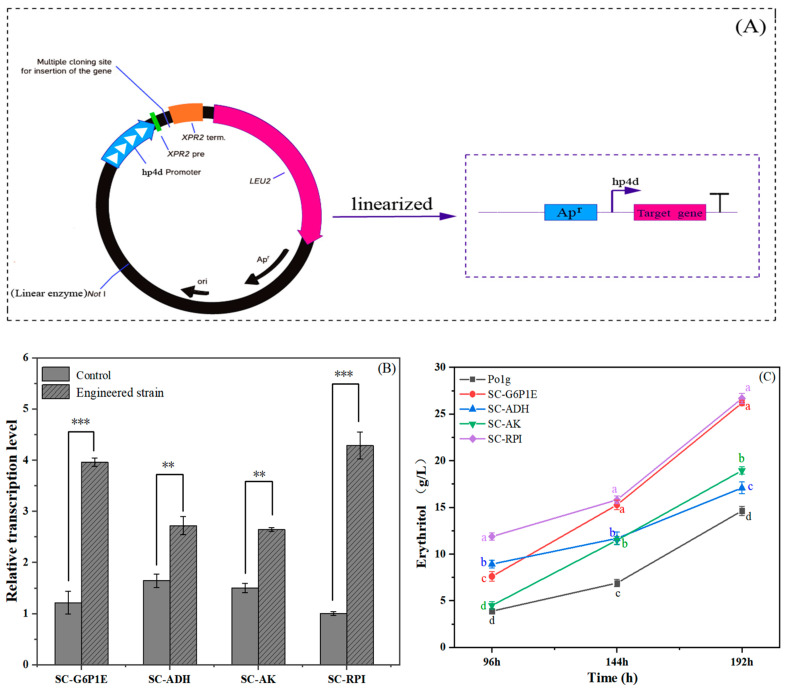
Improvement in erythritol production by rational metabolic engineering. (**A**) Construction of recombinant plasmids. (**B**) Relative transcript levels of the target gene in the engineered strain. Note: Data are presented as mean ± SD (n = 3). Statistical significance was determined by Student’s *t*-test (** *p* < 0.01; *** *p* < 0.001). (**C**) Time-course of the engineered strains for erythritol production, in which erythritol production was determined in the engineered strains containing genes encoding RPI, G6P1E, AK, and ADH. Note: Values labeled with different lowercase letters (a, b, c, d) indicate significant differences (*p* < 0.05, one-way ANOVA, biological replicates n = 3).

Figure 5 indicates the effects of individually overexpressing the genes encoding RPI, G6P1E, AK, and ADH in *Y. lipolytica* Po1g on erythritol production. Erythritol production in the engineered strains was evaluated through 250 mL shake-flask fermentation containing 30 mL of the initial fermentation medium, in which *Y. lipolytica* Po1g was used as a control strain. As shown in Figure 5, all the engineered strains produced a higher yield of erythritol when compared with the control strain *Y. lipolytica* Po1g, suggesting the significance of the gene expression level in the erythritol synthesis pathway for the highly efficient production of erythritol. After culturing for 192 h, the erythritol production in the engineered strain SC-RPI was 26.7 g/L, which exceeded that of the control strain *Y. lipolytica* Po1g by over 80%. The engineered strain SC-G6P1E maximally produced erythritol at a concentration of 26.2 g/L, which was marginally lower than that of the engineered strain SC-RPI. In contrast, the erythritol yield was greatly increased when compared with the wild-type strain *Y. lipolytica* Po1g, suggesting the significant contribution of G6P1E to erythritol synthesis in *Y. lipolytica*. So, an enhancement in the rate-limiting stage of the PPP is likely to facilitate its activation and increase the output of related metabolites. Engineered strains SC-AK or SC-ADH, overexpressing gene AK or ADH, exhibited an increased erythritol production of 18.9 g/L or 17 g/L, respectively, indicating that these two genes enhance erythritol biosynthesis. The data in Figure 5 indicate that the increasing extent of erythritol production for the engineered strains SC-AK and SC-ADH was smaller than that for the engineered strains SC-RPI and SC-G6P1E. This was presumably due to the overexpression of genes in the engineered strains SC-RPI and SC-G6P1E being directly related to the PPP, but genes in the engineered strains SC-AK and SC-ADH were excluded from the PPP. Thus, the up-regulation of key genes in the PPP presumably plays a more dominant role in erythritol production than that in the non-PPP, which further revealed that the PPP acted as a metabolic pathway for erythritol production. Hence, up-regulating the expression level of genes in the PPP is able to direct more carbon flux into the PPP, which is favorable for erythritol production [66]. These results illustrate the efficacy of the transcriptomics guided reverse metabolic engineering strategy, confirming the critical role of key genes in enhancing erythritol production in the mutant C1 strain and elucidating molecular mechanisms underlying improved biosynthesis with carbon flux redistribution. Additionally, we observed that the engineered strain exhibited higher erythritol production than the model strain but remained lower than the mutant, which may be attributed to multifaceted biological constraints. As mutant C1 may possess a more optimized transcriptional regulatory network than the targeted gene overexpression in the engineered strain, the mutagenesis process likely fine-tuned global regulators to achieve dynamic resource allocation irreplicable through synthetic pathway engineering. Moreover, long-term adaptive evolution during the serial plate passaging of the mutant strain may have introduced alterations rarely addressed in metabolic engineering. These discrepancies highlight the necessity of integrating systems biology with mutagenesis and laboratory evolution to bridge the gap between rationally designed strains and evolutionarily optimized mutants.

### 2.7. Influence of Culture Conditions on Erythritol Production

**Figure 6:**
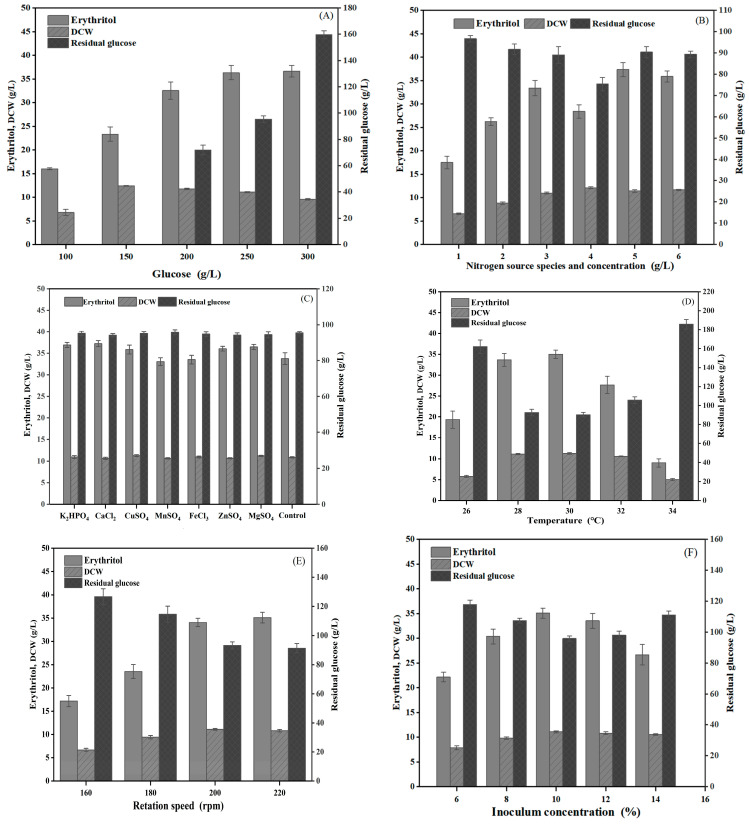
Effect of glucose content (**A**), yeast extract content or combined yeast extract with urea, where 1: 8 g yeast extract, 2: 10 g yeast extract, 3: 12 g yeast extract, 4: 15 g yeast extract, 5: 10 g yeast extract and 1 g urea, and 6: 10 g yeast extract and 1.5 g urea (**B**), metal ions (**C**), culture temperature (**D**), rotation speed (**E**), and inoculum concentration (**F**) on erythritol production by *Y. lipolytica* mutant C1.

The effect of the glucose content on erythritol production was determined, as shown in Figure 6A. Augmenting the glucose content gave rise to an elevated yield of erythritol, and the maximal erythritol production was obtained when mutant C1 was incubated in the medium containing 250 g/L and 300 g/L glucose. Previous studies have demonstrated that the high osmotic stress induced by elevated glucose concentrations plays a pivotal role in enhancing polyol biosynthesis in yeast [68]. Hence, this was presumably attributed to the enhanced osmotic pressure rise by the high concentration, which is capable of enhancing the fermentation effect. And as shown in Figure 6A, when the glucose concentration reached 300 g/L, erythritol production exhibited only marginal increases with substantial residual glucose. This suggests that excessive osmotic pressure inhibited microbial growth, consequently reducing glucose utilization. Moreover, elevated glucose concentrations could reduce dissolved oxygen in the fermentation broth, which is unfavorable for erythritol biosynthesis [49]. Consequently, 250 g/L glucose was preliminarily selected as the optimal concentration.

When the yeast extract was determined for erythritol production by *Y. lipolytica* mutant C1, the maximal erythritol yield was detected at an initial content of 12 g/L, as shown in Figure 6B. Brabender [69] investigated the potential of urea as a nitrogen source for *Y. lipolytica*, demonstrating that under identical cultivation conditions and C/N ratios, a urea-supplemented group achieved higher biomass and target product yields compared to an inorganic nitrogen source group ((NH_4_)_2_SO_4_). Consequently, this study employed a combination of urea and yeast extract as the nitrogen source for erythritol fermentation. Compared with a single nitrogen source, higher erythritol production was obtained when the yeast extract was combined with urea as the nitrogen source (Figure 6B).

It has been reported that some metal ions show an influence on polyol production due to an impact on enzyme activity implicated in polyol synthesis [70]. The introduction of KH_2_PO_4_ into the culture medium could promote erythritol production by *Y. lipolytica* mutant C1 to a large extent (Figure 6C). Nambou et al. proposed that a limitation in phosphate has a favorable impact on the accumulation of metabolic substances by *Y. lipolytica*, but phosphate interferes with the modulation of chemical oxidative stress stimulated by mineral elements, bringing about a delay in cell growth [71]. Magnesium ions can enhance erythritol production because they have been reported to be involved in a variety of life functions and enzymatic responses [71]. Lee et al. confirmed that both Mn^2+^ and Cu^2+^ can markedly increase erythritol yield because Mn^2+^ influences cell permeability and Cu^2+^ augments intracellular reductase activity [72]. In contrast, our results indicated that the addition of Mn^2+^ did not increase erythritol production, contrary to previous studies by Lee et al., and might be attributed to the distinct adaptations of different strains to metal ions. The data in Figure 6B also indicated that Ca^2+^ facilitated erythritol production, which is consistent with previous research [72].

The culture condition was optimized in the optimum culture medium. The influence of culture temperature on erythritol production is presented in Figure 6D, and the maximal erythritol production was obtained when *Y. lipolytica* mutant C1 was incubated at 30 °C. The rotational speed affecting erythritol production was also determined when culturing *Y. lipolytica* mutant C1 at 30 °C. When the rotational speed was over 200 rpm, *Y. lipolytica* mutant C1 maximally produced erythritol yield (Figure 6E). Meanwhile, higher erythritol production was achieved with inoculum sizes of 10% and 12% (Figure 6F).

### 2.8. Optimization of the Culture Medium Using Response Surface Methodology

Response surface methodology (RSM) has witnessed a proliferation in optimizing the components of microbial culture medium [44,73,74]. Given that polyol biosynthesis is influenced by diverse nutritional parameters in the culture medium, statistical optimization approaches, such as RSM, have been identified as an optimal strategy for refining culture conditions to enhance erythritol yields [75]. Considering that there are a large number of factors related to erythritol production, the Plackett–Burman (P-B) design was first employed to select the critical factors impacting erythritol production. The coding levels of the P-B experiment are provided in Table 1. As shown in Table 2, glucose, urea, and calcium chloride showed a considerably distinct influence on erythritol production (*p* < 0.05), whereas the other parameters did not manifest a significant effect *(p* > 0.05). Based on the P-B design experimental results in Table 2, a multi-response regression analysis was carried out by the utilization of a three-factor and three-level RSM to determine the combined effect of crucial variables in the culture medium. The coding levels for the Box–Behnken Design (BBD) experiment are provided in Table 3.

The result from a total of 15 experimental runs was obtained, as presented in Table 4. The observed value of erythritol production varied between 24.11 and 41.82 g/L at different experimental conditions. A subsequent test of significance in a quadratic regression equation by analysis of variance was carried out using Design Expert 12.0 software. As shown in Table 5, the *p*-values for the entire model stood at 0.0005, and those for lack-of-fit were 0.1780, which suggested that the experimental approach was reliable and showed a vigorous linear correlation. Moreover, the lack-of-fit measure (4.78) did not significantly deviate from the pure error, and the F value was 36.66, which further confirmed the correspondence and accuracy between the experimental data and the model prediction. The coefficient of determination (R^2^ = 0.9851) denoted that the model accounts for 98.51% of the variance in the response value, and the adjusted R^2^ of 0.9852 validated the robustness of the model. After quadratic regression fitting, the simulation equation amongst three factors and the erythritol production was developed as “Y = 43.36 + 6.19 X_1_ − 2.76 X_2_ + 1.83 X_3_ + 1.26 X_1_X_2_ − 3.05 X_1_X_3_ + 0.6452 X_2_X_3_ − 10.98A X_1_^2^ − 7.63 X_2_^2^ − 5.36C X_3_^2^”, where Y: response variable (erythritol yield), X_1_: glucose concentration, X_2_: urea concentration, X_3_: CaCl_2_ concentration, X_1_X_2_: interaction effect between glucose and urea concentrations, X_1_X_3_: interaction effect between glucose and CaCl_2_ concentrations, X_2_X_3_: interaction effect between urea and CaCl_2_ concentrations, X_1_^2^: effect of glucose concentration on Y, X_2_^2^: effect of urea concentration on Y, and X_3_^2^: effect of CaCl_2_ concentration on Y.

**Figure 7:**
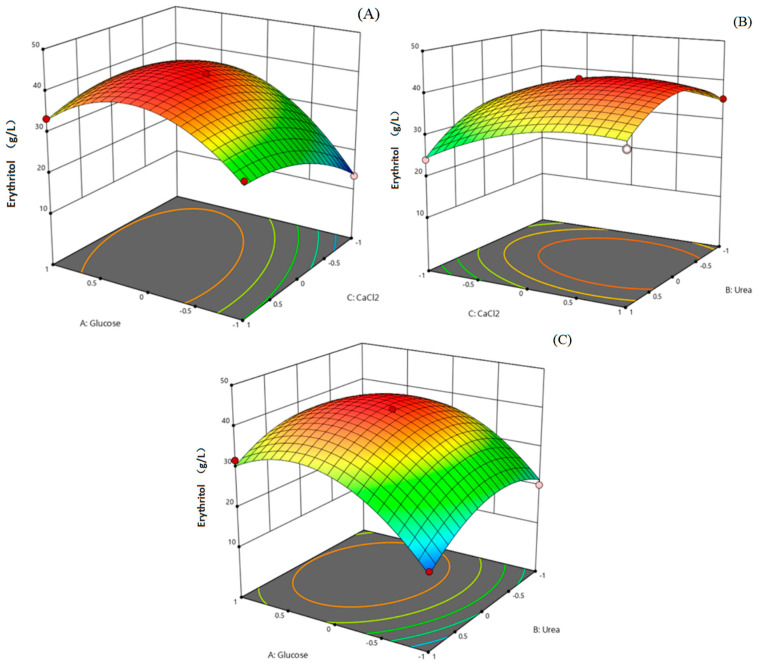
The combined effects of all factors on erythritol production analyzed by the response surface methodology. (**A**) The combined effect of glucose with CaCl_2_ on erythritol yield. (**B**) The combined effect of urea with CaCl_2_ on erythritol yield. (**C**) The combined effect of urea with glucose on erythritol yield. The color gradient in the three-dimensional response surface plot represents the magnitude of the response variable (erythritol production, g/L). As shown in the color bar, blue correspond to lower values, while red indicate higher values.

The variables were expressed as their coded values in the equation. The response surface plot delineating the collective effect of all factors on erythritol production is shown in Figure 7. According to the analysis using Design Expert 12.0 software, the maximum yield of erythritol was up to 54.6 g/L when the concentration of glucose, urea, and calcium chloride was fixed at 280 g/L, 1.021 g/L, and 7.32 g/L, respectively, in a 50 mL shake flask containing 15 mL culture medium for 192 h, and the erythritol production increased by 24% compared with the unoptimized medium. Hence, optimizing the medium components using RSM is an effective method for enhancing erythritol production, which aligns with previous studies [75].

### 2.9. Evaluation of Erythritol Production by Incubating Mutant C1 in a 10 L Fermenter

**Figure 8:**
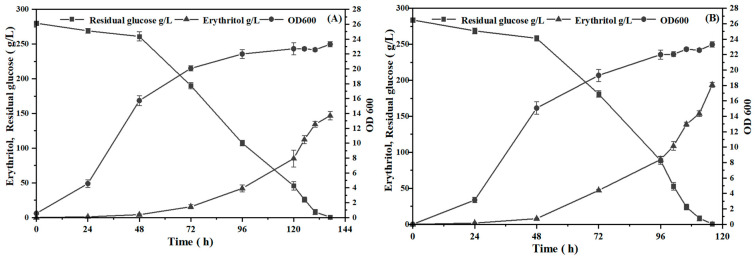
Time course of erythritol production and cell growth when *Y. lipolytica* of the wild-type strain (**A**) and mutant C1 (**B**) was incubated in a 10 L fermenter containing 7 L of the initial fermentation medium.

To evaluate the potential of erythritol production by *Y. lipolytica* mutant C1, batch fermentation was carried out in a 10 L fermenter with 7 L of the culture medium. Variable ventilation quality and stirring speed were used in the process of fermentation at 30 °C without adjusting the culture pH. As shown in Figure 8, a low ventilation quality and stirring speed resulted in rapid cell growth but a low erythritol production during the first 48 h of incubation. Such low erythritol production might be due to much more carbon flux being directed toward cell growth rather than erythritol synthesis in the beginning stage of fermentation. Subsequently, the accumulation of sugar alcohol becomes faster due to the increase in ventilation volume and stirring speed. As shown in Figure 8A, the erythritol content in the culture fluid was 149.33 g/L, and the productivity was 1.09 g/L/h after the wild-type strain was incubated for 137 h. In contrast, the erythritol content in the culture fluid was up to 194.47 g/L and the erythritol productivity was 1.68 g/L/h when mutant C1 was incubated for 116 h (Figure 8B). In comparison with the wild-type strain, the erythritol yield by *Y. lipolytica* mutant C1 was increased by 30%, and the fermentation time was shortened by 21 h.

Urea, as a nitrogen source, offers advantages of high nitrogen utilization efficiency and cost-effectiveness in maintaining strain metabolic activity. However, its application in industrial fermentation is constrained by pH fluctuations, elevated exhaust gas treatment costs, and regulatory limitations on urea residues in food products. In contrast, ammonium citrate, despite its higher material costs, ensures a stable ammonium ion supply, reduces downstream processing steps, and produces non-toxic metabolic by-products, thereby aligning with food-grade industrial requirements. Although optimized culture media could enhance erythritol production in mutant strains, this study prioritizes fermentation process optimization to improve erythritol yield, laying the groundwork for industrial scale-up. Consequently, the ammonium citrate-based initial medium was selected for 10 L fermenter fermentation following a comprehensive evaluation of industrial feasibility.

Fermentation occurred in a 10 L fermenter under optimized process conditions. As shown in Figure 8, the initial carbon source concentration in the 10 L fermenter (260 g/L) exceeded that in the initial fermentation medium due to the addition of 10% inoculum. During this fermentation process, given that *Y. lipolytica* can catabolize erythritol upon carbon exhaustion in the medium [76], a rigorous monitoring approach with increased sampling frequency during the late fermentation phase was implemented to ensure accurate quantification of the maximum erythritol production capacity of the strain. Compared with the wild-type strain, mutant C1 exhibited a nearly 30% increase in erythritol production alongside a reduced fermentation duration, demonstrating the higher productivity of mutant C1, which ranks competitively in analogous production systems [77]. Furthermore, mutant strain C1 exhibited minimal batch-to-batch variability in erythritol production, demonstrating its robust performance as a stable and efficient producer while suggesting its potential for industrial-scale biosynthesis. If the strain maintains this stability during scale-up processes and is combined with process optimizations such as dynamic feeding strategies in production, it will indicate significant market competitiveness and hold the potential to further reduce erythritol production costs.

## 3. Materials and Methods

### 3.1. Strain and Culture Conditions

The *Y. lipolytica* CICC 33063, obtained from the China Center of Industrial Culture Collection (CICC), served as the parental strain for mutagenesis. The auxotrophic strain *Y. lipolytica* Po1g (Leu^−^, Yisheng Biotechnology Co., Ltd., Taiwan Province, China) was utilized as the host strain. The *Y. lipolytica* LX-01 (mutant C1), deposited at the China Center for Type Culture Collection (CCTCC) under the accession number CCTCC NO: M 20221124, was generated through mutagenesis in this study. Yeast peptone dextrose medium (YPD), containing (in liters) 20 g glucose, 20 g peptone, and 10 g yeast extract, was used for culturing *Y. lipolytica* (pH = 6). The Triphenyltetrazolium chloride (TTC) medium consisted of (in liters) 200 g glucose, 15 g yeast extract, 0.1 g TTC, and 20 g agar, with the pH not manually adjusted. The high osmotic pressure screening medium, containing (in liters) 200 g glucose and 15 g yeast extract, was used for mutant screening, with the pH not manually adjusted. The initial fermentation medium was recommended by Qiu [78], with slight modifications. The initial fermentation medium was used for shake-flask fermentation and mRNA-Seq, containing 260 g/L glucose, 8 g/L yeast extract, 5 g/L ammonium citrate, 0.5 g/L MgSO_4_, 1.0 g/L KH_2_PO_4_, and 0.05 g/L CuCl_2_, with the pH not manually adjusted. For screening *Y. lipolytica* transformants, yeast nitrogen base (YNB) solid medium containing (in liters) 1.5 g yeast nitrogen base without amino acid and ammonium sulfate, 10 g glucose, 5 g (NH_4_)_2_SO_4_, and 20 g agar was used. The optimized erythritol fermentation medium for erythritol production in the fermenter comprised (in liters) 280 g glucose, 10 g yeast extract, 1.021 g urea, 1 g MgSO_4_, 2 g KH_2_PO_4_, 7.32 g CaCl_2_, 0.1 g CuCl_2_, and 0.1 g ZnSO_4_, with the pH not manually adjusted. Luria–Bertani (LB) medium was applied for the cultivation of *Escherchia coli*, in which Ampicillin (Amp, 0.1 g/L) was added as necessary (pH = 7). All media mentioned above were sterilized by autoclaving (SX-500, TOMY Corporation, Tokyo, Japan) at 115 °C for 30 min.

### 3.2. Mutagenesis and Screening

*Y. lipolytica* CICC 33,063 was incubated in YPD medium for 18–22 h at 30 °C with shaking at 200 rpm (ZWY-2102C; Beijing Kepusen Technology Co., Ltd., Beijing, China). After centrifugation at 5000× *g* for 10 min at 4 °C (5804 R; Eppendorf AG, Hamburg, Germany). The cultured cells were washed with 50 mL sterile saline 3 times and suspended in the same volume of sterile saline. For UV mutagenesis, 8 mL of the culture suspension was placed in a sterilized 90 mm Petri dish and exposed to a 20 W UV lamp (distance: 20 cm) for varying durations ranging from 0 to 110 s with 10 s intervals. Subsequently, the mutated cells were spread on the TTC plate, and the positive mutant was selected by red colony, as described by Ghezelbash et al. [17]. After that, the positive mutant derived from UV treatment served as the new parental strain for ARTP mutagenesis by previous guidelines with slight modifications [44]. Specifically, 10 μL of cell suspension was applied onto a sterilized stainless-steel plate connected to an ARTP mutation breeding device (Siqingyuan Biotechnology Co., Ltd., Beijing, China). The sample was subjected to helium plasma exposure lasting between 60 and 200 s, while measurements were taken every increment of thirty seconds. The temperature at the plasma torch nozzle was at 30 °C with a consistent distance of 2 mm between the steel plate and the helium plasma source. Upon completion of mutagenesis, mutants collected from metal plates were diluted in sterile saline and spread onto a TTC medium plate for assay. Both the colony count and lethality rate curves were recorded.

The large and red single colonies on the TTC plate were selected and inoculated into a 24-well plate containing high osmotic pressure screening medium and incubated at 30 °C and 200 rpm for 96–144 h. The culture fluid was centrifuged at 5000× *g* for 10 min, and the positive mutants producing erythritol were selected visually by thin layer chromatography (TLC) on a Merck TLC plate (Merck KGaA, Darmstadt, Germany), as described previously, with slight modifications, in which the potassium permanganate was used as chromogen [79]. The erythritol produced by positive mutants was quantified using a high-performance liquid chromatography (HPLC) system (Agilent 1260 Infinity II, Agilent Technologies, Santa Clara, CA, USA) equipped with a refractive index detector (RID) maintained at 35 °C. Separation was performed on an Agilent Zorbax NH2 column (4.6 × 250 mm, 5 μm) held at 35 °C, with an isocratic mobile phase of acetonitrile/water (8:2, *v*/*v*) at a flow rate of 1.0 mL/min. The injection volume was 10 μL, and the elution time was set to 30 min. The outstanding mutant was evaluated for genetic stability by conducting ten generations and assessing the erythritol production of each generation.

### 3.3. Bioassay for Biomass Quantification

For biomass concentration determination, 500 μL of the fermentation broth was centrifuged at 5000× *g* for 10 min, washed twice with deionized water, and appropriately diluted. The optical density (OD 600) was measured at 600 nm using a UV-Vis spectrophotometer (Model UV-2000, UNICO Instruments, Dayton, NJ, USA). Cell dry weight (DCW) was determined by drying the harvested biomass at 80 °C to a constant weight.

### 3.4. Transcriptome Analysis

To elucidate the mechanism responsible for the augmented erythritol production, three biological replicates of each sample were utilized for the transcriptome sequencing of the mutant and the wild-type strain. The wild-type strain and mutant C1 were cultured in the initial fermentation medium and incubated at 30 °C and 200 rpm for 96 h. The culture fluid was centrifuged at 4000× *g* for 10 min to collect the cells. RNA sequencing libraries were prepared by Beijing Novogene Co., Ltd. (Beijing, China). Raw sequencing data underwent quality filtering before gene expression quantification using RSEM expectation maximization with FPKM normalization [80]. Differentially expressed genes (DEGs) were identified through comparative analysis with significance thresholds set at padj ≤ 0.05 and |log 2Fold Change (FC)| ≥ 1 [81].

### 3.5. Real-Time Quantitative PCR Analysis

Total RNA was isolated from wild-type and mutant strains using an RNA extraction kit (Accurate Biotechnology Co., Ltd., Hunan, China). First-strand cDNA was synthesized from 1 μg of total RNA using the kit (Accurate Biotechnology Co., Ltd., Hunan, China). Quantitative real-time PCR (qPCR) was performed on a CFX Opus 96 Real-Time PCR System (Bio-Rad Laboratories, Hercules, CA, USA) with gene-specific primers (Supplementary Table S1). The relative mRNA levels were quantified through the 2^−ΔΔCT^ method with *ACT1* as an internal reference [82]. The primers used in this study are provided in the Supplementary Materials (Table S1)

### 3.6. Rational Metabolic Engineering of Y. lipolytica Po1g

Based on transcriptome analysis, *Y. lipolytica* Po1g was selected as the chassis strain for rational metabolic engineering. Total RNA was extracted from *Y. lipolytica* Po1g using an RNA extraction kit and reverse-transcribed into cDNA. Target genes were amplified using cDNA as the template. The target gene fragments and plasmid pYLSC (Yisheng Biotechnology Co., Ltd., Taiwan Province, China) were double-digested with SfiI/XbaI and SfiI/HindIII, respectively, to generate compatible cohesive ends. The digested fragments were ligated into plasmid pYLSC using T4 DNA ligase, positioning the target gene downstream of the strong hybrid promoter hp4d and secretion signal peptide (XPR2), while placing it upstream of the XPR2 transcriptional terminator (Figure 5A). The recombinant plasmids were transformed into *Escherichia coli* DH5α cells for propagation. Following sequencing validation, the plasmids were linearized with NotI and electroporated into *Y. lipolytica* Po1g competent cells [83]. Transformants were selected on yeast nitrogen base (YNB) plates lacking leucine, as the plasmid carries a functional LEU2 gene to complement the leucine auxotrophy of the host strain *Y. lipolytica* Po1g (Leu⁻), thereby restoring prototrophic growth. The successful integration of the target gene into the host genome was achieved by PCR with universal primers specific to the plasmid. To quantify the transcriptional levels of target genes in the engineered strains, qPCR was performed, with *Y. lipolytica* Po1g serving as the control.

### 3.7. Optimization of Culture Conditions

Mutant C1 was cultured in a 50 mL shake flask with 15 mL culture fluid to examine the medium composition and culture conditions for promoting erythritol production. The medium constituents were optimized by means of a one-factor-at-a-time method. Specifically, the glucose content (100, 150, 200, 250, and 300 g/L), the yeast extract content (8, 10, 12, and 15 g/L), the yeast extract (10 g/L) combined with urea (1 and 1.5 g/L), and ions, including K⁺ (2 g/L), Mg^2^⁺ (1 g/L), Ca^2^⁺ (4 g/L), Cu^2^⁺ (0.1 g/L), Mn^2^⁺ (0.1 g/L), Fe^3^⁺ (0.1 g/L), and Zn^2^⁺ (0.1 g/L), were selected for optimization. After the culture medium was optimized, the culture parameters, including temperature and rotation speed, were fine-tuned in the optimized medium. Based on the obtained one-factor optimization for the component of the culture medium, a P-B experiment was conducted to select the significant factors capable of enhancing erythritol production, and 3 dummy variables were introduced to assess the standard deviation of the model. Design Expert 12 software was employed to evaluate the significance level of each factor for identifying those exerting a substantial influence on the response value. Subsequently, the BBD of RSM was utilized to construct a statistical model for determining the individual and interactive effects of those significant factors chosen based on the P-B test of the fermentation process.

### 3.8. Batch Fermentation in a 10 L Fermenter

Batch fermentation was carried out in a 10 L fermenter (BLB10-108J, Shanghai Bailun Biotechnology Co. Ltd., Shanghai, China) with 7 L of the initial fermentation medium at 30 °C without adjusting the culture pH, in which both ventilation quantity and agitation speed were variable in the diverse phases of the culture process. In the first 24 h of fermentation, the ventilation quantity was set at 0.6 m^3^/h, and the agitation speed was set at 200 rpm. In the period of 24–36 h, the ventilation quantity was set at 0.7 m^3^/h, and the agitation speed was set at 250 rpm. In the period of 36–48 h, the ventilation quantity was at 0.8 m^3^/h, and the agitation speed was at 300 rpm. When beyond 48 h, both the ventilation quantity and agitation speed were set at 1.6 m^3^/h and 600 rpm, respectively. The culture was sampled at 24 h intervals to detect the erythritol content and biomass.

### 3.9. Statistical Analysis

All tests were performed in triplicate, and the results were expressed as mean and standard deviation (SD), unless otherwise stated. Analysis of variance and significant differences among means were tested by the independent-sample *t*-test (*p* < 0.05) using SPSS software (version 25.0, SPSS Inc., Chicago, IL, USA), as necessary.

## 4. Conclusions

Mutant C1, with high erythritol productivity and robust genetic stability, was obtained by the combination of ARTP and UV mutagenesis. Key genes playing a critical role in improving erythritol production were identified via comparative transcriptomics and further validated through RT-qPCR. The individual overexpression of key genes was shown to enhance erythritol production via metabolic engineering. It was presumed that the redistribution of carbon flux and the supply of precursor substances play a crucial role in improving erythritol production. The batch fermentation in a 10 L fermenter indicated that the erythritol production by mutant C1 was 194.47 g/L, and the productivity was 1.68 g/L/h, whereas the erythritol yield by the wild-type strain was 149.33 g/L, and the productivity was 1.09 g/L/h. Compared to the wild-type, mutant C1 showed a 30% increase in erythritol production and an 21 h reduction in fermentation time. These findings provide a foundation for enhancing erythritol production through metabolic engineering and synthetic biology strategies, with potential for industrial-scale applications.

## Figures and Tables

**Table 1:** The coding levels of the P-B experiment.

		Levels (g/L)	
Code	Type	−1	+1
X_1_	Glucose	125	250
X_2_	Urea	1	1.5
X_3_	Glycine	2	5
X_4_	KH_2_PO_4_	1	2
X_5_	MgSO_4_	1	1.25
X_6_	CaCl_2_	3	6
X_7_	CuSO_4_	0.1	0.125
X_8_	ZnSO_4_	0.1	0.125

**Table 2:** Statistical analysis of the P-B design.

Source	Sum of Squares	df	Mean Square	F-Value	*p*-Value
Model	759.76	8	94.97	13.99	0.0264
X_1_(Glucose)	532.97	1	532.97	78.53	0.0030
X_2_(Urea)	96.93	1	96.93	14.28	0.0325
X_3_(Glycine)	4.72	1	4.72	0.6957	0.4654
X_4_(KH_2_PO_4_)	38.73	1	38.73	5.71	0.0969
X_5_(MgSO_4_)	3.41	1	3.41	0.5030	0.5293
X_6_(CaCl_2_)	76.50	1	76.50	11.27	0.0438
X_7_(CuSO_4_)	0.0436	1	0.0436	0.0064	0.9411
X_8_(ZnSO_4_)	6.47	1	6.47	0.9532	0.4009
Residual	20.36	3	6.79		
Cor Total	780.12	11			

**Table 3:** Coding levels for the BBD experiment.

Variables	Coding Levels		
	−1	0	+1
A (Glucose)	125	250	375
B (Urea)	0	1	2
C (CaCl_2_)	4	7	10

**Table 4:** The BBD experimental results.

Experimental Number	A (Glucose)	B (Urea)	C (CaCl_2_)	Predicted Value (g/L)	Experimental Value (g/L)
1	0	0	0	41.36	40.23
2	−1	1	0	15.30	16.05
3	0	0	0	41.36	41.82
4	0	−1	−1	31.08	32.83
5	1	0	−1	34.05	33.05
6	0	−1	1	34.94	35.34
7	−1	0	−1	13.83	13.48
8	0	1	−1	24.52	24.11
9	−1	0	1	28.08	29.08
10	0	0	0	41.36	42.02
11	1	0	1	33.10	33.45
12	−1	−1	0	21.57	20.17
13	1	1	0	30.43	31.83
14	1	−1	0	31.68	30.93
15	0	1	1	33.96	32.21

Note: Predicted value represents results obtained by software simulation. Experimental value represents real experimental results.

**Table 5:** The BBD experimental model of influencing factors.

Source	Sum of Squares	DF	Mean Square	F	Prob (P) > F	Significance
Model	1036.74	9	115.19	36.66	0.0005	***
A (Glucose)	318.53	1	318.53	101.38	0.0002	
B (Urea)	28.39	1	28.39	9.03	0.0299	
C (CaCl_2_)	88.51	1	88.51	28.17	0.0032	
AB	6.30	1	6.30	2.01	0.2159	
AC	57.76	1	57.76	18.38	0.0078	
BC	7.81	1	7.81	2.49	0.1757	
A^2^	386.76	1	386.76	123.09	0.0001	
B^2^	150.16	1	150.16	47.79	0.0010	
C^2^	54.93	1	54.93	17.48	0.0086	
Residual	15.71	5	3.14			
Lack of Fit	13.79	3	4.60	4.78	0.1780	ns
Pure Error	1.92	2	0.9620			
Cor Total	1052.45	14				
R^2^_Adj_	0.9582					
R^2^_Pred_	0.7863					
R^2^	0.9851					
CV%	5.82					

Note: *** *p* < 0.001; ns: not significant; CV%: Coefficient of Variation

## Data Availability

Raw sequencing data: NCBI BioProject. Accession: PRJNA975267. Reviewer link: https://dataview.ncbi.nlm.nih.gov/object/PRJNA975267?reviewer=dk6gcbbvlept2emnmguh3er56l. Release Date: 9 October 2025. The generated data are publicly available immediately, if necessary.

## Supplementary Materials

The following supporting information can be downloaded at https://www.mdpi.com/article/10.3390/ijms26094180/s1.
