# Supplementary material for: Comparative Transcriptome Analysis Reveals Key Genes Related to Erythritol Production in Yarrowia lipolytica and the Optimization of Culture Conditions
Source: Int J Mol Sci. 2025 Apr 28;26(9):4180. doi: 10.3390/ijms26094180 (PMC12071344; doi:10.3390/ijms26094180)
Supplement: Supplementary file 1 [file ijms-26-04180-s001.zip › ijms-3503643-supplementary.pdf]

Table S1. Primers used in this study.

Primers	Sequence
EryA-qRT-F	TTGTGCCGAATCAATCCCGA
EryA-qRT-R	AGGTAGGGCTCGATGGACTT
EryB-qRT-F	CCACACCTATCTGCGAGTCC
EryB-qRT-R	TGACAGCTCCAGGCACATTT
EryC-qRT-F	CTCCGAACTGCCTCCTCTTG
EryC-qRT-R	TTCTCGGCAACGTAGACCAC
EryD-qRT-F	GGAATCACCAACCAGCGAGA
EryD-qRT-R	GTTGGAGGTGTCGGTGATGT
EryE-qRT-F	ACCCGAGCTGGTAAGGATCT
EryE-qRT-R	TGTCGTAGGCAGTGGTGAAC
EryF-qRT-F	CCAAGTGCAAGAACGGCTTC
EryF-qRT-R	GACCTTGTGGTAGGATCGGC
EryG-qRT-F	TGAGCTGGGTCACGGATCTA
EryG-qRT-R	CGAGTTGGGGGTGTTGATGA
EryH-qRT-F	AGGACTCGGAACACTTGCTG
EryH-qRT-R	GGCAGACCGACCAAAACAAC
EryI-qRT-F	CGAGTTTGAGCGAGTCCGAT
EryI-qRT-R	TTGAAGTTGGGCTCTCGCTT
ACT1-qRT-F	CTGGCACCACACCTTCTACA
ACT1-qRT-R	TCTCTCGGTTGGACTTGGG
SC-G6P1E-F	GCGGCCGTTCTGGCCATGTCCGTGTCCCAAACCGA
SC-G6P1E-R	GCTCTAGACTACAAATGAGCCTTGATCT
SC-RPI-F	GCGGCCGTTCTGGCCATGTCCTCCGAACTGCCTCC
SC-RPI-R	GCTCTAGATTACTTGGTAATGGTGGAGA
SC-AK-F	GCGGCCGTTCTGGCCATGTCCACCATTGAAGATCT
SC-AK-R	GCTCTAGATCAATCATCTCCTTCTTGGG
SC-ADH-F	GCGGCCGTTCTGGCCATGTCTGCTCCCGTCATCCC
SC-ADH-R	GCTCTAGA TTACTTGGAGGTGTCCAGAA
